# Next-Gen Therapeutics: Pioneering Drug Discovery with iPSCs, Genomics, AI, and Clinical Trials in a Dish

**DOI:** 10.1146/annurev-pharmtox-022724-095035

**Published:** 2024-12-17

**Authors:** Zehra Yildirim, Kyle Swanson, Xuekun Wu, James Zou, Joseph Wu

**Affiliations:** 1 Stanford Cardiovascular Institute and Division of Cardiovascular Medicine, Stanford University School of Medicine, Stanford, California, USA;; 2 Department of Computer Science, Stanford University, Stanford, California, USA; 3 Greenstone Biosciences, Palo Alto, California, USA

**Keywords:** iPSC, clinical trials in a dish, drug discovery, cardiotoxicity

## Abstract

In the high-stakes arena of drug discovery, the journey from bench to bedside is hindered by a daunting 92% failure rate, primarily due to unpredicted toxicities and inadequate therapeutic efficacy in clinical trials. The FDA Modernization Act 2.0 heralds a transformative approach, advocating for the integration of alternative methods to conventional animal testing, including cell-based assays that employ human induced pluripotent stem cell (iPSC)-derived organoids, and organ-on-a-chip technologies, in conjunction with sophisticated artificial intelligence (AI) methodologies. Our review explores the innovative capacity of iPSC-derived clinical trial in a dish models designed for cardiovascular disease research. We also highlight how integrating iPSC technology with AI can accelerate the identification of viable therapeutic candidates, streamline drug screening, and pave the way toward more personalized medicine. Through this, we provide a comprehensive overview of the current landscape and future implications of iPSC and AI applications being navigated by the research community and pharmaceutical industry.

## INTRODUCTION

**Figure 1:**
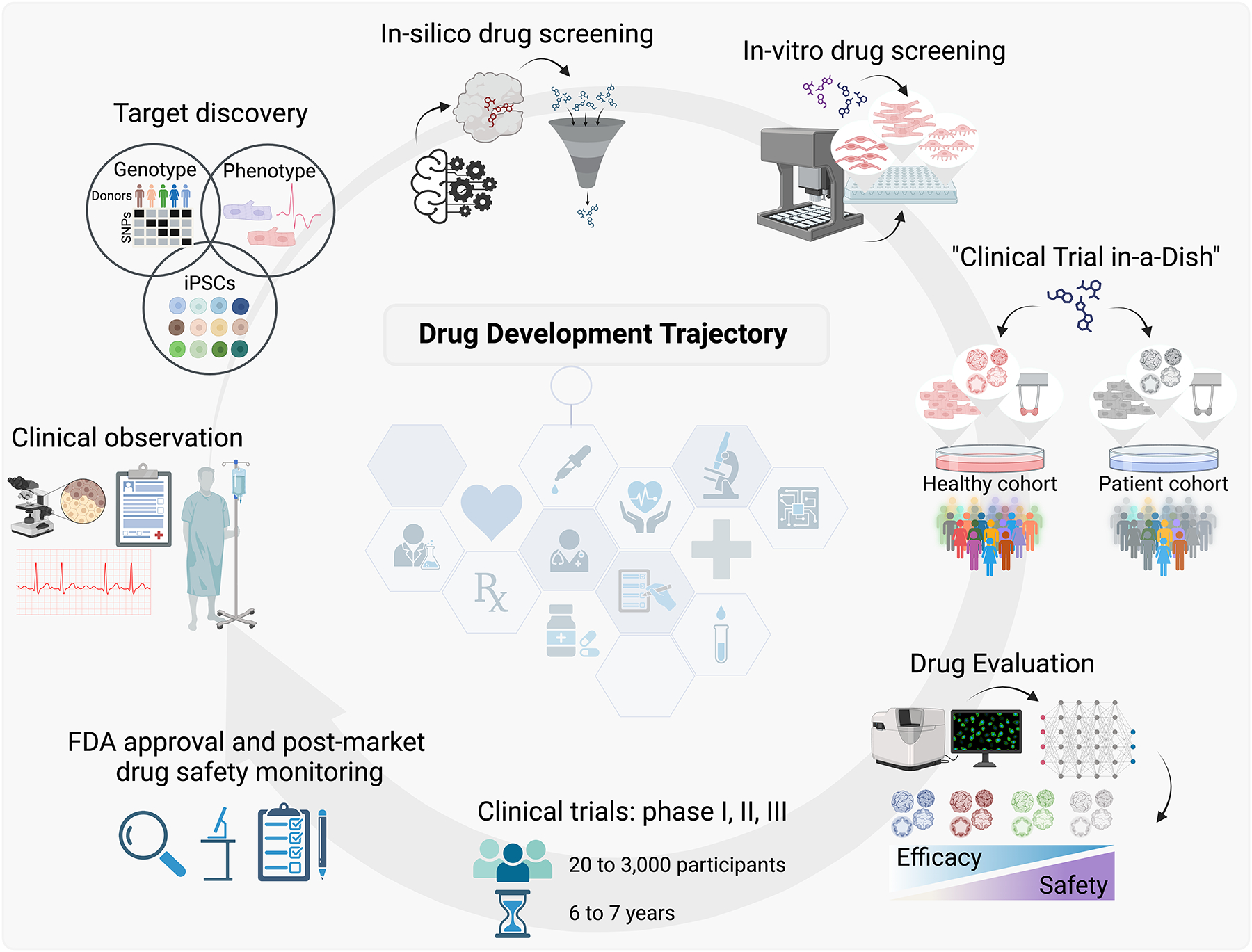
Artificial intelligence–enhanced induced pluripotent stem cell (iPSC) drug discovery. During the foundational stage of drug discovery and development, scientific inquiry begins with clinical insights into disease processes and meticulous investigations of the underlying molecular mechanisms. Utilizing state-of-the-art technologies, researchers inspect a multitude of compounds to unearth those with the potential capability to alter disease progression. In preclinical research, selected compounds undergo rigorous testing, including studies in cell cultures and clinical trials in a dish, to assess their safety, biological activity, and potential efficacy. Promising candidates advance to clinical trials, where they are tested in human volunteers or patients under strictly regulated conditions. Upon successful clinical trials, the drug is submitted for US Food and Drug Administration (FDA) review, where it is rigorously evaluated. After a drug is approved and marketed, it is continuously monitored for any long-term or rare side effects through various postmarket safety surveillance mechanisms. Figure adapted from images created with BioRender.com.

The journey of bringing a new drug to market is a complex and multiphased process, encompassing a series of critical steps that ensure safety and efficacy. Modern drug development typically begins with discovery and development, where new compounds are identified through insights into disease processes or the recognition of potential therapeutic effects of existing treatments. This stage is followed by preclinical research, where compounds showing promise undergo rigorous testing in laboratory and animal studies to assess their safety profile and biological activity. Next, during clinical research, compounds that have demonstrated potential in preclinical studies are tested in human subjects across several phases of clinical trials to evaluate their efficacy, safety, and appropriate dosage. Successful candidates then proceed to regulatory authorities such as the US Food and Drug Administration (FDA) for drug review, where they undergo a detailed examination by regulatory authorities. Finally, after a drug enters the market, it is still subject to FDA postmarket drug safety monitoring to track any long-term or rare side effects that may not have been evident during the trial phases (1, 2) (Figure 1).

Preclinical research stands as the bedrock of drug discovery and development, aimed at unveiling therapeutics that not only pledge efficacy but also uphold stringent safety standards. In the United States alone, approximately $28 billion is spent on preclinical research annually (3). However, the transition from preclinical research to human clinical application has a success rate below 10%, predominantly due to unforeseen toxicities and a lack of therapeutic efficacy not anticipated by traditional preclinical animal models (4–6).

**Figure 2:**
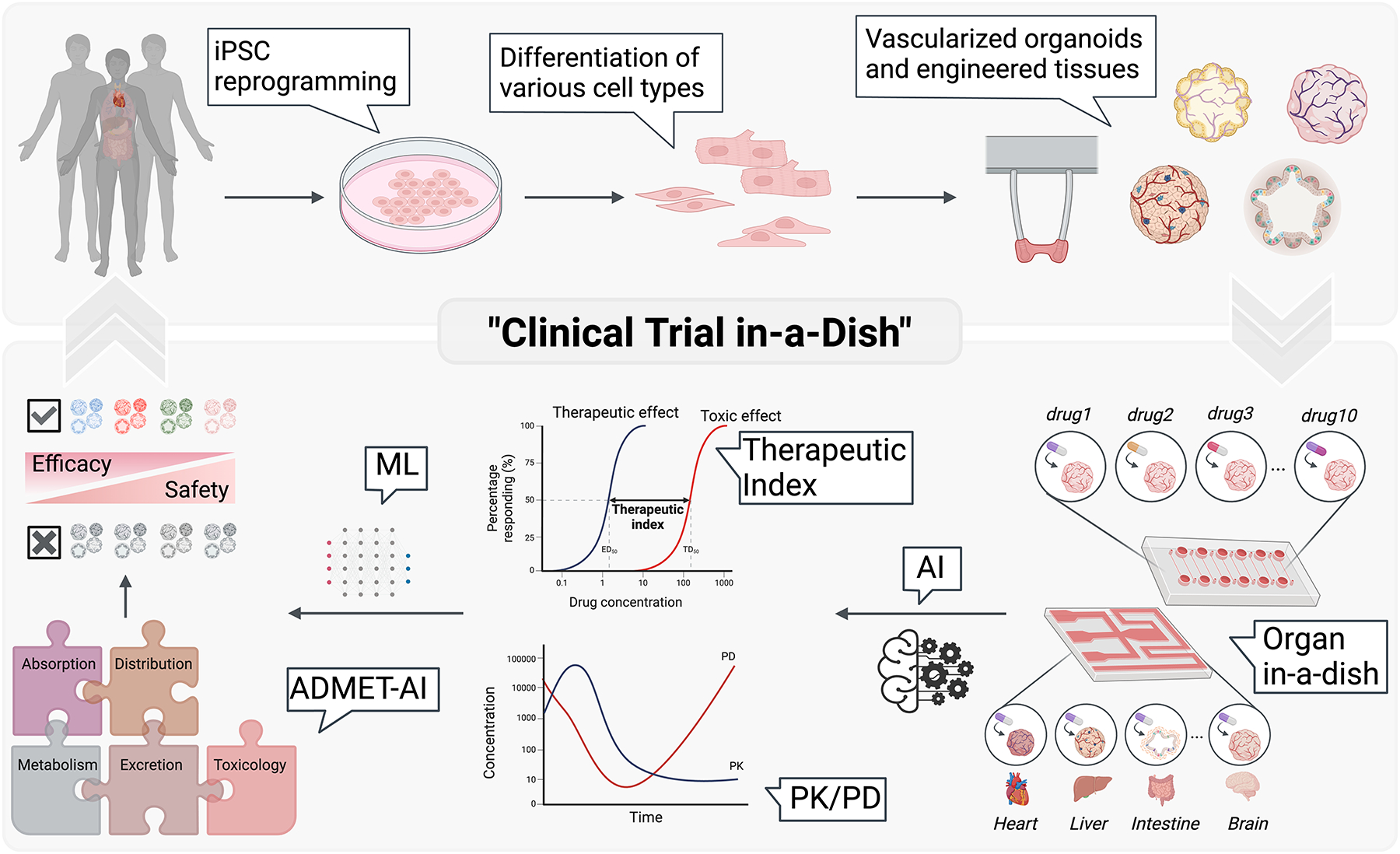
Clinical trials in a dish. More advanced drug evaluation begins with the reprogramming of somatic cells into induced pluripotent stem cells (iPSCs), which are then differentiated into cardiac cells. These cells are used to construct vascularized organoids and engineered tissues, which are integrated into organ-on-a-chip systems. Within this microengineered environment, sophisticated in vitro pharmacodynamics (PD) and pharmacokinetics (PK) analyses are conducted, leveraging artificial intelligence (AI) and machine learning (ML) to predict absorption, distribution, metabolism, excretion, and toxicity (ADMET) outcomes. This high-throughput screening and evaluation method assesses drug safety and efficacy, directly informing patient-specific therapeutic strategies. Figure adapted from images created with BioRender.com.

With the FDA Modernization Act 2.0 coming into effect in December 2022, a new chapter in scientific advancement has arrived to tackle ethical considerations in animal testing. This law advocates for the adoption of alternative, nonanimal methods such as cell-based assays leveraging human induced pluripotent stem cells (iPSCs), organoids, and organ-on-a-chip technologies, as well as progressive artificial intelligence (AI) technology (7–9). Our review explores the influence of these advancements on drug discovery and development, pivoting from traditional practices to more predictive, ethically aligned methodologies, particularly the pioneering clinical trials in a dish concept (Figure 2).

## DRUG DISCOVERY AND DEVELOPMENT: HARNESSING OPPORTUNITIES AND OVERCOMING CHALLENGES

The drug development process is an intricate and phased journey, with meticulously designed phases to evaluate a drug candidate’s safety, efficacy, pharmacokinetics (PK), and pharmacodynamics (PD). Safety screening during the early stages serves as a prophylactic measure aimed at eliminating compounds with potential adverse effects before they progress to costly clinical trials. Identifying safety concerns early not only protects patient health but also ensures the efficient allocation of health-care resources.

### Drug Development: Ensuring Safety, Proving Efficacy, and Securing Market Success

Despite continuing advances, the path to clinical translation faces many hurdles. A significant number of new drugs fail human clinical trials due to an increased risk of adverse effects such as ventricular arrhythmias unforeseen in the preceding cellular and animal study phases. Approximately 30% of potential therapeutics are terminated early in the development process due to concerns surrounding cardiotoxicity and efficacy (10, 11). Moreover, compounds that pass initial safety assessments in Phase I trials frequently face withdrawal from the market later after the discovery of unanticipated toxicities during subsequent Phase II and III trials, which have failure rates of 22% and 35%, respectively (12).

These challenges highlight the need to navigate the intricate complexities of cardiovascular diseases (CVDs), which necessitates innovative approaches in drug discovery. CVDs remain a leading cause of death globally, with diverse manifestations that culminate in heterogeneous responses to treatment among individuals (13). In addressing the management of specific cardiac conditions, it is crucial to distinguish between diseases with genetic determinants, such as hypertrophic cardiomyopathy (HCM) and transthyretin amyloid cardiomyopathy (ATTR-CM), and more common multifactorial conditions. While targeted therapies for genetically defined diseases such as HCM and ATTR-CM are emerging that offer tailored treatment based on mechanistic understanding (14, 15), the management of common cardiovascular diseases remains challenging due to the complex interplay of genomics, environmental influences, and aging (16, 17).

The persistent reliance on animal models for drug development and safety screening has long been problematic, primarily due to their inability to consistently or accurately replicate human physiological responses. This discrepancy can result in the misprediction of human-specific toxicities and efficacies of drugs. Such limitations have contributed to an overall failure rate exceeding 90% over the past three decades (18). The complexity of this challenge is further amplified in the context of CVDs, where pronounced discrepancies between species in cardiac physiology can skew results based on animal models (19, 20). Onakpoya et al. (21) reported that from 1953 to 2013 a total of 462 drugs were withdrawn from the market, with cardiovascular complications accounting for 63 withdrawals. Premarket screening techniques predominantly identify only the most common toxicities that manifest within short time frames, thus missing many undetected adverse reactions. The retrospective analysis of drug withdrawals over six decades underscores the gravity of postmarketing safety concerns and stresses the need for more sophisticated and predictive preclinical and clinical screening methods.

### Opportunities Abound: The Dawn of AI and Human iPSC Models in Drug Discovery for Cardiovascular Diseases

AI has begun to support the drug discovery process across four key domains: (*a*) target selection and indications, where AI facilitates the discovery of novel gene-disease linkages crucial for identifying new therapeutic targets; (*b*) lead matter identification and optimization, through efficient design, screening, and optimization of therapeutics; (*c*) clinical translation, by using data to enhance insights that streamline and expedite clinical trials; and (*d*) operational efficiency, by refining end-to-end operations to accelerate the delivery of therapies to the market.

Building on these capabilities, the employment of AI in drug discovery is advancing the identification of novel biomarkers and drug targets. By integrating structural biology with expansive multiomics data, generative AI applications significantly contribute to the process of preclinical evaluations. Computational approaches combine pharmacophore screening (22), reverse docking (23), and structure similarity assessments (24) to improve biomarker identification (25, 26); design of drug-like molecules (27); investigation of absorption, distribution, metabolism, excretion, and toxicity (ADMET) property predictions (28); and the prioritization of indications (29), all of which bolster the compilation and interpretation of vast data sets to enhance the architecture of in silico drug screening and trial design (30). Although still in nascent stages, AI-derived therapeutics such as glucagon-like peptide 1 receptor (GLP1R) for type 2 diabetes and obesity, apelin receptor (APLNR) for pulmonary arterial hypertension and idiopathic pulmonary fibrosis, Rho-associated coiled-coil-containing protein kinase 1/2 (ROCK1/2) for diabetic complications, and sphingosine-1-phosphate 1 (S1P1) for postmyocardial infarction care are making headway in clinical research (31). As a result, drug development is increasingly predictive and congruent with the overarching objectives of precision medicine of curtailing adverse drug reactions and optimizing patient-centric care.

Furthermore, the progress in human iPSC technologies, alongside breakthroughs in gene editing and screening technologies such as CRISPR-Cas9 (32), as well as the deepening understanding derived from genomic and proteomic profiling (33), is setting the stage for what may be a fundamental transformation of drug safety screening and development in CVD in the foreseeable future. Induced pluripotent stem cell–derived cardiomyocytes (iPSC-CMs) may represent a pivotal application of iPSC technology within cardiovascular research (34). Unlike primary cardiomyocytes, which are limited by postmortem viability and abbreviated culture longevity, iPSC-CMs offer a dynamic and sustainable platform. These cells are a potent asset in modeling, as they closely emulate the pharmacological responses of human cardiomyocytes. iPSC-CMs accurately replicate cardiac properties to provide a more relevant model for primary cardiac function, thereby offering novel insights into genetic variants responsible for disease phenotypes (35). While still in the nascent stages, these tools have the potential to address some of the longstanding challenges in the field (36).

## ADVANCING DISEASE MODELING WITH iPSCs FOR TARGET DISCOVERY

iPSC technology has influenced studies of cellular and tissue models, particularly for cell types that were previously challenging to access or propagate in vitro (37). Reprogramming somatic cells, such as skin fibroblasts or peripheral blood mononuclear cells, into iPSCs is now a streamlined process leveraging the transformative potential of Yamanaka factors [Octamer binding transcription factor 3/4 (OCT3/4), SRY-box transcription factor 2 (SOX2), Krueppel-like factor 4 (KLF4), and cellular myelocytomatosis oncogene (c-MYC)] delivered via nonintegrating methods such as Sendai virus vectors (37, 38). These iPSCs are characterized by their immortality and clonal expansion capabilities, thus providing a sustainable source of cells for diverse applications (39–41). Meticulously designed protocols leading to various cell types enable the differentiation of specific cardiac cells and their subtypes, which foster the development of sophisticated cardiac models (42–53). These iPSC-derived cardiovascular cells, cultured under controlled conditions, offer a dynamic and versatile platform for drug discovery, screening, and disease modeling.

### Disease Modeling: Replicating Pathophysiological Conditions In Vitro

iPSCs retain the unique genetic profiles of their donors, making them invaluable tools for investigating the underlying mechanisms of complex CVDs such as dilated cardiomyopathy (DCM). iPSC-CMs harboring mutations in key cardiac-related genes, including titin (*TTN*) (54), lamin A/C (*LMNA*) (55), myosin heavy chains 6 and 7 (*MYH6*, *MYH7*) (56), sodium channel protein type 5 subunit alpha (*SCN5A*) (57), and myosin-binding protein C (*MYBPC3*) (58), have provided significant insights into the molecular underpinnings of cardiac function and disease. For instance, *LMNA* mutation–bearing iPSC-CMs have demonstrated critical nuclear irregularities, enhanced apoptotic activity, and abnormal electrophysiological behaviors, affirming the capability of iPSC models to accurately reflect the intricate nature of cardiac diseases (55).

### Gene Editing: Tailoring Cellular Genomics for Targeted Research

iPSCs offer a unique advantage by allowing for the introduction of precise genetic variations with the use of advanced genome editing techniques such as CRISPR-Cas9, making it possible to study rare genetic variations that are otherwise challenging to capture from patients (59). By editing a healthy control iPSC line, researchers can bypass the lengthy process of patient recruitment and cell reprogramming, vastly expediting and economizing the research process (39). For instance, cardiomyocytes with engineered *TTN* truncation mutations have demonstrated phenotypic hallmarks of DCM, such as compromised sarcomere function and stress response, and reduced growth factor signaling, mirroring the disease phenotype seen in patient-derived iPSC-CMs (54). This method has proven that such genome-edited models can effectively mimic the disease phenotype. Additionally, correcting pathogenic mutations in diseased iPSCs provides an opportunity to deepen our understanding of monogenic diseases and their underlying mechanisms (60).

### Organoids: Recapitulating Organ Complexity in the Lab

While useful for many disease modeling and screening assays, 2D iPSC cultures are limited by their inability to replicate 3D environments of live organ systems. This results in reduced cellular heterogeneity and a more embryonic phenotype that resembles early developmental stages rather than adult mature tissues (61, 62). To address these limitations, researchers have engineered various methods to coax cellular maturity in vitro. Techniques such as extended culture periods, the introduction of oxidative stressors, the shift to adult metabolic profiles, and the expression of aging-associated proteins like progerin have been applied to model adult-onset diseases, including Parkinson’s disease and arrhythmogenic right ventricular dysplasia/cardiomyopathy (ARVC/D) (63–65). However, these strategies have their own pitfalls, as they may artificially accelerate aging or maintain an inherent immaturity that could distort the true nature of disease mechanisms.

Organoids provide a significant advancement in biomedical research, offering 3D, self-organizing cultures that closely replicate the architecture and functionality of human organs (66, 67). These miniature yet complex models help bridge the gap between traditional 2D cell cultures and whole-animal systems, providing a more physiologically relevant context for studying human development, disease pathology, and drug efficacy. The advent of 3D culture systems offers a promising avenue, providing a milieu that supports enhanced maturation and more accurately recapitulates the development of relevant cardiovascular lineages under study (68). The pioneering work by Sato et al. (69) established the foundation for organoid technology with the cultivation of the first organoids derived from mouse small intestine stem cells. This breakthrough involved the meticulous reconstruction of the intestinal epithelial niche in vitro, enabling the growth of single cells into complex 3D aggregates composed of various cell types, mirroring the intricate microarchitecture of the natural intestinal environment (69, 70).

iPSC-derived cardiac models have emerged as advanced tools for mirroring the complex physiology of both normal and pathological human hearts. Their versatility allows for the organization of cardiac cells into 3D tissues, including organoids, spheroids, and engineered tissue constructs. Such 3D models offer a dynamic approach to recapitulate cellular diversity and cardiac functions, providing a more physiologically relevant environment for developmental studies and toxicity assessments (71–76). For instance, healthy iPSC lines, devoid of genetic anomalies, yield tissues suitable for investigating the impact of environmental factors and assessing drug toxicity. These cardiac organoids are particularly sensitive to external stimuli, which can significantly affect cell behavior such as proliferation and differentiation pathways (77). Cardiac organoids have also proven useful in understanding cardiac development and metabolic diseases linked to congenital heart defects. For instance, they have successfully modeled heart defects induced by pregestational diabetes, demonstrating that organoids can indeed mimic in vivo conditions (66, 78). Manipulating pathways such as wingless/int-1 (WNT) signaling via glycogen synthase kinase 3 (GSK3) inhibition has led to the creation of self-organizing heart organoids that exhibit a diverse composition of cardiac cell lineages and robust beating activity, akin to that of a developing fetal heart (66). Furthermore, techniques such as high-throughput organoid screening have shed light on the induction of adult sarcomeric proteins and the suppression of cell proliferation, which are fundamental to cardiac maturity (79).

### Omics-Based Clinical Discovery: Harnessing Genomics, Transcriptomics, Epigenomics, and Proteomics in iPSC-Derived Models

In the realm of precision medicine, the integration of omics technologies with iPSC platforms is essential for a detailed exploration of disease mechanisms, genetic variability, and individual therapeutic responses (80). This approach facilitates the mapping of complex biochemical pathways and genetic networks, which is critical for developing targeted treatments (81).

#### Genomics.

In contemporary genomics, DNA sequencing techniques have been used to delineate a spectrum of genetic variations, including single-nucleotide polymorphisms, insertions, deletions, and gene copy number variations. These variations are pivotal for dissecting the genetic determinants of diseases and unique human characteristics. Genome-wide association studies (GWASs) are another principal research tool used to identify genomic variants associated with disease susceptibility or specific phenotypic traits. In CVD research, GWASs have elucidated numerous genetic loci linked to coronary artery disease (CAD) to highlight its multifaceted genetic etiology. A broad GWAS encompassing 181,522 CAD cases revealed 241 genetic associations, with a cross-ancestry analysis identifying over 80 novel loci. Functional validation using CRISPR-Cas9 technology demonstrated the role of a regulatory enhancer in RhoGAP myosin IXb (MYO9B), providing insights into its influence on CAD through vascular cell behavior (82). Another extensive evaluation of 36 studies with 743,919 CAD cases identified ancestry-specific loci, emphasizing the necessity for and value of diverse population representation in GWASs to fully understand CAD’s genetic landscape and pathophysiology (83). While further replication and validation studies are essential, these findings underscore the importance of inclusive genomic research in uncovering the complete genetic architecture of CAD.

Furthermore, genomic tools have significantly enhanced population-level studies, facilitating the identification of individual donors in large-scale drug toxicity screenings and cell village models (84, 85). Critical to this effort are biobanks such as the Stanford Cardiovascular Institute Biobank (86), which maintain extensive collections of iPSC lines, each accompanied by comprehensive whole-genome sequencing and clinical data. Biobanks allow researchers to trace genetic lineage within a multiplexed screening platform, elucidate disease mechanisms, and develop therapies tailored to specific genetic profiles.

#### Transcriptomics and epigenomics.

These fields are crucial for understanding cellular function, disease etiology, and therapeutic effects, thereby helping to realize the goals of precision medicine. Technological advances such as single-cell RNA sequencing and single-cell assay for transposase-accessible chromatin using sequencing have significantly enhanced our ability to analyze transcriptomic and epigenomic landscapes. In particular, the single-cell/nuclei technology has been pivotal in studying the human heart’s development and organization, emphasizing the role of various cardiac cell types. The heart’s architecture includes cardiomyocytes, which are vital for the heart’s biochemical, mechanical, and electrical functions; pacemaker cells and Purkinje fibers that manage electrical impulses; and supportive cells such as endocardial, endothelial, and smooth muscle cells, as well as epicardial-derived fibroblasts, all collaborating to maintain effective cardiac output (87–92). These studies have enriched our knowledge of the healthy versus developing versus failing human heart, refining transcriptional signatures specific to cardiomyocyte subtypes, and establishing a foundation for their derivation from iPSCs. This increasingly detailed understanding of gene signatures across cardiomyocyte subtypes aids in the differentiation and maturation of specific cell types from iPSCs, significantly improving our ability to use iPSC-CMs (93–95).

Building on detailed knowledge about the cells of the heart, these methods have been instrumental in understanding cardiotoxic effects of immunosuppressants such as tacrolimus and sirolimus through studies on iPSC-derived cardiac organoids, which reveal specific fibrotic pathways influenced by these drugs (96). Moreover, the integration of these technologies with bioreactor-facilitated mass production of organoids offers a robust platform for drug and cardiotoxicity evaluations (97).

#### Proteomics and metabolomics.

The iPSC technology coupled with proteomics and metabolomics has advanced the understanding of disease mechanisms and drug discovery, facilitating the identification of proteins and metabolites critical to essential biological pathways (80, 98). As an example, the integration of mass spectrometry-based metabolomic and proteomic analyses has enhanced the resolution at which we can observe the molecular landscape of end-stage kidney disease (ESKD) patients undergoing hemodialysis. This comprehensive workflow has uncovered an array of elevated molecules persisting in the serum after dialysis, such as lipids that pinpoint issues such as suboptimal filtering efficiency and selectivity of the treatment. The elucidation of these molecules, particularly those entwined with CVD, which represent a significant mortality risk factor in ESKD, has been corroborated through validation using human iPSC-derived cardiomyocytes (99).

## FROM IN SILICO TO IN VITRO: SYNERGIZING TECHNOLOGIES FOR ROBUST DRUG DEVELOPMENT

Over the past decade, AI has transformed health care by processing extensive physiological data to generate actionable medical insights, among other achievements (100, 101). Integration of AI with iPSC techniques is helping to make personalized medicine possible by facilitating the transition from in silico predictive models to in vitro applications. AI plays a crucial role in improving in silico drug screening processes by improving the design and evaluation of drug candidates. It helps refine safety profiles based on iPSC-derived data and supports the in silico design of novel compounds for subsequent validation using iPSC assays. This combined approach leverages the strengths of both AI in silico modeling and the biological relevance of iPSCs to expedite the progression from theoretical models to practical, clinically relevant applications.

### Pioneering Predictive Analytics: AI for In Silico Drug Screening

AI-driven in silico drug screening holds great promise. After identifying promising molecules via clinical observations and iPSC assays, AI algorithms can be used to forecast a compound’s pharmacological potential, scrutinizing attributes such as toxicity and bioavailability before in vivo application. This predictive approach represents a substantial leap beyond traditional experimental or animal testing, offering a more efficient and cost-effective pathway by preemptively identifying possible shortcomings of drug candidates.

AI methods are essential for analyzing molecular characteristics by utilizing digital representations of compounds to make predictive assessments. Traditional models such as SwissADME (102) and DeepTox (103) utilize calculated molecular descriptors—such as molecular weight or logP—to inform AI-driven models for predicting essential ADMET properties. Contemporary approaches increasingly adopt graph neural networks (GNNs) (104), which conceptualize molecules as graphs, with atoms and bonds corresponding to nodes and edges, respectively. GNNs synthesize basic atomic and bond features to form AI-generated descriptors that are refined to enhance prediction accuracy for various molecular properties (105, 106), including ADMET (28). These methods critically evaluate potential drugs identified through iPSC-based research based on molecular structures, which can then be re-evaluated on iPSC platforms for efficacy and toxicology assessments. Resulting lead candidates are analyzed using algorithms such as GNNs, along with machine learning models like random forests to predict PD and PK. As an example, this approach has been used to integrate AI-predicted in vivo rat PK properties with ADMET forecasts to infer human in vivo PK profiles, enhancing drug development accuracy and decreasing the use of a large number of animals (107).

Despite the potential benefits of AI in drug discovery, several operational considerations and potential drawbacks need to be acknowledged. Firstly, the deployment of these AI methods requires significant computational power, often necessitating advanced graphics processing units and substantial memory capacities to handle the large data sets typical in biomedical research. The input data for these systems may include high-dimensional biological data sets such as genomic sequences, chemical structures, or high-resolution imaging data (108, 109). Moreover, there are inherent limitations and challenges associated with the data used to feed the AI. One major constraint is the quality and diversity of the data used: AI models are only as good as the data they train on. Inadequate or biased data can lead to overfitting, wherein models perform well on training data but poorly on unseen data, or to the propagation of existing biases, leading to skewed results. On a more fundamental level, the black box nature of many AI systems can impede the understanding of how decisions are made, complicating the validation of AI-driven hypotheses against experimental or clinical outcomes (110, 111). Additionally, the overreliance on extensive computational resources can incur high operational costs and energy demands, limiting the accessibility of AI technologies for some research institutions or smaller enterprises. These factors demand a balanced approach that considers the transformative potential of AI in enhancing drug discovery alongside the practical challenges and ethical considerations involved in its implementation.

### AI-Driven Compound Discovery and Validation Using iPSC Assays

AI has expanded its reach into the drug discovery process, being used not only in evaluating molecules from experimental drug screens but also in identifying potential hits through AI-based virtual screening. Furthermore, a new breed of AI known as generative models can now directly design hit compounds, foregoing the need to screen a vast virtual library. Subsequently, these AI-identified or AI-invented compounds undergo empirical validation using iPSC-based assays.

#### AI-based virtual screening.

AI-based virtual screening employs molecular property prediction models to appraise potential compounds within a virtual library. For instance, Stokes et al. (112) utilized a GNN named Chemprop (106) to anticipate antibacterial activity in molecules. This AI system was trained with a data set of known antibacterial agents and subsequently applied to predict the efficacy of thousands of compounds. A substantial number of these AI-predicted molecules exhibited the desired biological activity, showcasing AI’s ability to efficiently navigate and refine extensive virtual libraries that dramatically surpasses conventional experimental screening throughput. In the realm of target-based drug discovery, AI models can integrate drug and protein target data. DeepDTA (113) exemplifies this approach by applying convolutional neural networks (CNNs) to predict protein-ligand binding affinities using simplified molecular-input line-entry system strings for drugs and amino acid sequences for proteins. The advent of 3D protein structures from the Protein Data Bank (114) and AI-predicted models such as AlphaFold (115) has further enhanced the accuracy of these AI systems. Methods such as DiffDock (116) employ diffusion models to dock compounds to protein targets, further refining the process of target-specific virtual screening.

#### AI-based molecule design.

Generative AI models are transforming the traditional screening process by efficiently designing molecules tailored for specific targets, thereby reducing the extensive evaluation of large compound libraries. For example, the fragment-based generative AI model by Powers et al. (117) incrementally adds molecular fragments to optimize protein pocket binding. Similarly, the multi-objective GFlowNet (118) learns to create molecules that simultaneously satisfy multiple criteria such as protein binding and drug-like properties. Generative AI models, while efficient, may design complex molecules that are challenging to synthesize. To address this, Swanson et al. (119) developed SyntheMol, which navigates a vast chemical space of easily synthesizable molecules to propose viable antibiotic candidates. Their approach not only expedites the design of potential drugs but also ensures that these molecules can be readily synthesized and validated, including through iPSC-based assays.

In conclusion, AI can enhance the early stages of drug discovery by both providing high-throughput screening capabilities and facilitating the design of novel compounds. After these AI-generated molecules are identified, iPSC-based assays provide the empirical validation needed to confirm their therapeutic potential, thus accelerating the transition from theoretical design to clinical application.

### Optimizing iPSC and Organoid Drug Screening with AI: Advancing Toward Predictive and Personalized Therapeutics

By integrating AI’s advanced analytical capabilities, researchers can boost the accuracy and efficiency of both image- and measurement-based screenings. This synergy facilitates the identification of nuanced biological responses to pharmaceutical compounds, tailoring treatments to individual genetic and phenotypic profiles, and spearheading a more targeted and effective approach to therapeutic development.

#### Image-based analysis.

Following the establishment of cell-based assays, AI enhances the interpretative analysis by scrutinizing morphological changes or quantifying assay measurements. AI models adeptly analyze morphological alterations in iPSC-derived cells after drug application, detecting subtle yet significant patterns indicative of the drug’s impact. For instance, Grafton et al. (62) trained a CNN to discern cardiotoxicity from iPSC-derived cardiomyocyte images. The training process involved exposing the CNN to cardiomyocytes altered by known cardiotoxic agents such as doxorubicin and contrasting these with images of untreated cells. This process trained the AI to accurately pinpoint morphological hallmarks of cardiotoxic effects. Upon deploying this CNN across a library of 1,280 bioactive compounds, the AI flagged several substances as cardiotoxic, some of which were FDA-approved drugs with documented cardiovascular concerns in clinical contexts. Such proficiency of AI in early-stage drug screening promises a more preemptive and comprehensive identification of cardiotoxic compounds, thereby improving the drug development process.

Organoids, as 3D biological models, can also be evaluated using AI-powered image-based assessments. AI-driven computational platforms often necessitate individual cell segmentation within organoids to measure and analyze cellular characteristics; thus, CNNs such as the U-Net architecture are being harnessed for this purpose (120, 121). CNNs like these can both segment cells and classify them into specific functional states based on staining patterns (e.g., identifying proliferative cells via Ki67 staining). Moreover, CNNs can evaluate entire organoid regions to categorize morphological (121) or genetic variances, such as distinguishing between normal versus Huntington’s disease–associated phenotypes (122). In drug discovery, such models are used to scrutinize morphological alterations induced by pharmacological agents within organoids, offering insights into both therapeutic effects and potential toxicities in a process that is guided by the observed morphological transformations.

#### Measurement-based analysis.

AI-based measurement analysis extends the reach of drug discovery beyond morphological assessments by focusing on the intricate functional changes within cells that may not be visibly apparent. AI algorithms can delve into the nuanced data collected via assays, such as the kinetic profiles of calcium transients in cardiomyocytes or the dose-response curves of cytokines in renal cells, to predict toxicity and other effects. For instance, conventional machine learning algorithms such as random forests, support vector machines, and *k*-nearest neighbors have been successfully applied to such data sets for toxicity predictions (36, 123). Another example involves the calcium transient signals in iPSC-CMs, which have been used to forecast cardiotoxicity, whereas cytokine response patterns in renal-like cells have been used to study nephrotoxicity (124–126). In more sophisticated applications, CNNs have been trained to analyze action potential waveforms in cardiomyocytes by classifying drug effects and even adjusting predictions based on drug dosage or specific pathogenic mutations induced by CRISPR-Cas9, thus showcasing their potential for personalized drug response analysis (36).

Moreover, AI’s potential is being explored by studies assessing the ability of drugs to rectify dysregulation across entire gene networks. A notable study employed targeted RNA sequencing of a gene panel in endothelial cells with a *NOTCH1* mutation, using AI to compare drug-induced gene expression changes with wild-type patterns, resulting in identifying effective compounds that could normalize the genetic network (127). These applications demonstrate how AI can provide a comprehensive analysis of diverse assay measurements, thereby enriching the drug screening process with iPSCs and offering a promising new avenue for discovering more targeted and effective therapeutics.

## FROM BENCH TO BEDSIDE: BRIDGING PRECLINICAL RESEARCH WITH CLINICAL TRIAL IN A DISH PARADIGMS

In the dynamic field of pharmaceutical development, the gap between preclinical research and clinical trials presents a significant challenge. Addressing this gap is crucial for drug development. The innovative approach of clinical trials in a dish refers to a cutting-edge method in medical research that utilizes advanced cell culture technologies to model human diseases and test therapeutic interventions within a controlled laboratory environment. This approach simulates the cellular and molecular aspects of human physiology on a microscale, allowing researchers to quickly observe the effects of drugs and other treatments in a human-like context before clinical trials. Overall, this method aims to improve the accuracy of preclinical studies, reduce the reliance on animal models, and tailor therapies to individual genetic profiles.

The integration of iPSC technology, advanced organoid science, and AI into the clinical trials in a dish concept represents a significant advancement by facilitating dynamic preclinical models (128–130). These models effectively mimic human pathophysiology and are specifically designed to predict individual patient responses (67, 76, 131–133). One example of this innovative concept is exemplified in the investigation of LMNA-related DCM, a condition stemming from mutations in the *LMNA* gene that encodes lamins A and C, known as cardiolaminopathy (128).

### Cell Village and Population-Scale Screening

One innovative use of iPSCs is the cell village in a dish concept, which uses these cells for population-scale studies. This model excels in analyzing the effect of complex interactions of genetic and environmental factors on cellular phenotypes, broadening our insights into disease mechanisms and therapeutic interventions (134). iPSCs sourced from a diverse array of individuals that reflect varied ethnic, racial, gender, and sexual identities are collectively differentiated and concurrently exposed to pharmacological assessments, streamlining the drug screening process. Such a strategy effectively minimizes batch discrepancies and the inherent random variations that typically arise from staggered testing procedures. Leveraging advanced multiomic platforms, this approach offers a refined scope to scrutinize drug-induced transcriptomic and epigenomic alterations. It establishes a scalable, precise, and ethically attuned structure that is suitable for broad-scale research, enriching our comprehension of the intricate interplay among genetics, environmental influences, and pharmacological treatments.

### Organoids in a Dish

Advancements in organoid technology have significantly propelled cardiac modeling in a dish development for drug discovery. Richards et al. (135) engineered cardiac organoids that incorporate an oxygen-diffusion gradient that mimics the human heart’s response to myocardial infarction when stimulated by norepinephrine. These organoids exhibited zonal variations akin to infarcted tissue, providing a detailed model for studying myocardial infarction hallmarks, including metabolic disturbances, fibrosis, and calcium dysregulation. Furthermore, these cardiac organoids showed an increased cardiotoxic response to doxorubicin under hypoxic conditions, indicating their value in simulating diseases with nongenetic factors and in drug testing. Current research is fine-tuning organoid-based methodologies to enhance drug safety and efficacy testing, cementing their vital role in medical breakthroughs and therapeutic development (136–139).

### Multiorgan Microphysiological Systems

While still in their nascent stages, microphysiological systems (MPSs) such as organ-on-a-chip devices are emerging as promising supplements to traditional animal models. These systems integrate living cells in engineered environments that replicate physiological fluid flows, providing a potentially more accurate model of organ-level physiology and disease. While organ-on-a-chip technology is still evolving, it represents a promising avenue that may complement traditional animal models by potentially reducing their use. Although not yet ready to fully replace animal testing, these MPSs are progressively refining our approach to simulating organ-level physiology and pathology, demonstrating significant potential for growth and utility in research and therapeutic development (140).

Researchers are working on MPSs that combine iPSC-based models with microfluidic technologies to create intricate models that emulate the interactions between diverse tissue types in a meticulously controlled setting. These platforms facilitate the cross-talk between the organoids, allowing for an in-depth investigation of human physiology and pathology and the assessment of pharmacological impacts under laboratory conditions (141, 142). The incorporation of iPSCs enhances the relevance of these systems, as they can generate patient-specific models that mirror genetic diversity and disease-specific phenotypes. When multiple organ-specific organoids are combined within a single MPS, this integration offers a more comprehensive representation of the complex interactions occurring within the human body. For example, MPSs that incorporate liver modules can provide critical insights into drug metabolism. These systems predict metabolites and their potential toxicity to inform optimal dosing strategies, increasing therapeutic efficacy while minimizing adverse reactions (141, 143).

The heart-on-a-chip represents a significant advancement in MPS technology, utilizing human iPSC-CMs to create laminar cardiac tissues on flexible extracellular matrix gels with embedded multielectrode arrays. This design facilitates real-time monitoring of tissue-level electrophysiological responses, contributing to the fields of disease modeling, drug discovery, and cardiotoxicity assessment (144–148). For instance, Zhang et al. (149) developed a heart-on-a-chip model using bioprinting to create tissues lined with endothelial cells that mimic blood vessels. This system, suitable for drug screening, showed cardiomyocytes and endothelial cells responding to varying drug doses.

Ronaldson-Bouchard et al. (150) showcased a multiorgan tissue chip system that offers a patient-specific platform for evaluating new treatments and drug toxicity markers. This system sustains the integrity of individual tissues while facilitating their interaction, which is crucial for human iPSC-derived tissues that are still maturing. The inclusion of an endothelial barrier and vascular flow in the design allows for natural cell responses, selective drug transport, and immune cell movement, with enhanced effects observed in more complex, multi-tissue chips compared to single-tissue setups.

## FUTURE DIRECTIONS AND POTENTIAL IMPACTS OF CLINICAL TRIALS IN A DISH ON THE PHARMACEUTICAL INDUSTRY

The advent of human iPSC technologies, coupled with strides in AI technology, promises an exciting frontier for the pharmaceutical industry. This synergy is poised to revolutionize drug discovery by fostering the development of personalized medications that are tailored to the genetic makeup of individual patients or specific patient subgroups. With iPSCs enabling more precise modeling of diseases and AI algorithms efficiently parsing through complex data sets, the industry anticipates marked improvements in the speed and accuracy of identifying viable drug candidates.

The integration of multiple organoid types within MPS platforms further underscores the potential for iPSCs to model intricate interorgan interactions. This could impact investigation and treatment for particularly intractable diseases that involve several bodily systems and for evaluating the efficacy of combinational therapies. From an economic perspective, the downstream effect could lead to a reduction in the overall cost of drug development, with savings potentially passed on to patients through more affordable medications.

As the pharmaceutical industry begins to embrace these innovative approaches, regulatory bodies will also need to evolve, such as by adopting new frameworks to accommodate the novel data streams from iPSC- and AI-driven research. Ultimately, this fundamental transformation of the drug development process promises to deliver safer and more effective therapeutic options, launching health care into a new era where meeting complex global health needs in a timely manner is more achievable than ever before.
